# Supplementary material for: Learning curve and surgical outcome of robotic assisted colorectal surgery with ERAS program
Source: Sci Rep. 2022 Nov 29;12:20566. doi: 10.1038/s41598-022-24665-w (PMC9709162; doi:10.1038/s41598-022-24665-w)
Supplement: Supplementary file 1 — Supplementary Information 1. [file 41598_2022_24665_MOESM1_ESM.zip › LSC Raw data-Ñ[▒K/085/P_ñw╝╨░O▒Kñσ.pdf]

[redacted]

Pathologic diagnosis:
1. Desending colon, laparoscopic anterior resection --- a. Adenocarcinoma, moderately to poorly differentiated, arising from background of tubulovillous adenoma with micropapillary feature. Tumor invaded through serosa with marked tortuosity. The bilateral cut ends and mesocolic lymph nodes (0/24) are free of tumor. b. Tubular adenoma with low grade dysplasia, 2 foci.
2. Left colic root, dissection --- Unremarkable lymph nodes (0/3) and fibrovasculoadipose tissue.

Ancillary study for diagnosis:
IHC stains for PMS2 and MSH6 are done on section H. Both markers are preserved.

Prognostic and predictive factor:
1. Tumor size: 8x5.5 cm.
2. Depth of invasion: Tumor invaded through the serosa with marked tortuosity.
3. Histologic grade: G2: Moderately differentiated to G3: Poorly differentiated.
4. Angiolymphatic invasion: Not identified.
5. Perineural invasion: Not identified.
6. Tumor deposits (discontinuous extramural extension): Not identified.
7. Macroscopic tumor perforation: Not identified.
8. Surgical Margins:
a) Proximal Margin: Uninvolved by invasive carcinoma, distance of tumor from margin: 7.5 cm.
b) Distal Margin: Uninvolved by invasive carcinoma, distance of tumor from margin: 10 cm.
c) Radial or Mesenteric Margin: Uninvolved by invasive carcinoma, distance of tumor from margin: 2.5 cm.
9. Lymph node status: Uninvolved (0/27).
10. Extranodal involvement: Not identified.
11. Tumor regression grading S/P CCRT: No known presurgical therapy.
12. Type of polyp in which invasive carcinoma arose: Not identified.
13. Additional pathologic findings: Adenomas.
14. Pathological TNM Stage: pT4aN0 (According to the eighth edition, American Joint Committee on Cancer Staging Guidelines for Tumors).
15. TNM descriptors: Not applicable.

Gross description:
The specimen consists of 1) a segment of colon, 17.5 cm in length and 4.5 cm in proximal cut end circumference, 4.6 cm in distal circumference, and mesocolic soft tissue. A gray tan firm annular elevated and centrally ulcerative tumor, 8x5x5.5 cm, is noted, 10 cm to the distal cut end. On cross section, the tumor invades through the serosa with inflammatory tract connecting to another part of mucosa (within the same segment), 2.5 cm to the radial margin, and serosal retraction is also noted. Two polyps, up to 0.8x0.7 cm, are also seen distal to the main tumor. The both cut ends are free of tumor grossly. 2) Left colic root, a piece of tan yellow soft tissue, 5.4x3.9x0.9 cm. Representative part for section: A) proximal cut end B) distal cut end C) polyps D-H, Q-T) tumor (Q-S: inflammatory tract through serosa, Q-R: mapped) J) radial margin K) omentum L) specimen 2 M-P) lymph nodes.

Note:
1. The interpretation of MMR panel and recommended studies are as below: No loss of nuclear expression of MMR proteins: low probability of microsatellite instability-high (MSI-H). Please note: There are exceptions to the above IHC interpretations. These results should not be considered in isolation, and clinical correlation with genetic counseling is recommended to assess the need for germline testing.
2. This case has been peer reviewed by two doctors.
#T-59461_2 #M-81403_2 3 1105 S0000F
#T-C4560_2 #M-09183_2 0 1105 000000

已電子簽章

敬告：本單僅供醫師參考使用，不具任何申請效力！！
請病患妥為保管，若不慎遺失需自負個人資料外洩責任！
